# Foot and Ankle Trauma: Epidemiology Before, During, and Post COVID-19 Pandemic in a Level I Trauma Center: A 5-Year Experience and Data Analysis

**DOI:** 10.3390/jcm13247585

**Published:** 2024-12-13

**Authors:** Antonio Mascio, Tommaso Greco, Chiara Comisi, Virginia Cinelli, Nicola De Gasperis, Marcello Candelli, Francesco Franceschi, Marcello Covino, Giulio Maccauro, Carlo Perisano

**Affiliations:** 1 Department of Orthopedics and Geriatric Sciences, Catholic University of the Sacred Heart, 00136 Rome, Italy; antonio.mascio87@gmail.com (A.M.); virginiacinelli23@gmail.com (V.C.); giulio.maccauro@policlinicogemelli.it (G.M.); carlo.perisano@policlinicogemelli.it (C.P.); 2 Department of Orthopedics and Rheumatological Sciences, Fondazione Policlinico Universitario A. Gemelli IRCCS, 00136 Rome, Italy; greco.tommaso@outlook.it; 3 Department of Life Sciences, Health, and Healthcare Professions, Link Campus University, 00165 Rome, Italy; n.degasperis@unilink.it; 4 Concordia Hospital, Via delle Sette Chiese, 90, 00145 Rome, Italy; 5 Department of Emergency Medicine, Fondazione Policlinico Universitario A. Gemelli, IRCCS-Catholic University of the Sacred Hearth, 00168 Rome, Italy; marcello.candelli@policlinicogemelli.it (M.C.); francesco.franceschi@policlinicogemelli.it (F.F.); marcello.covino@policlinicogemelli.it (M.C.)

**Keywords:** COVID-19, emergency department, foot, ankle

## Abstract

**Background**: Foot and ankle injuries are one of the leading orthopedic causes of emergency department (ED) admissions. The purpose of this study was to analyze, through 5 years of data collection, differences in number and type of admissions, as well as the severity of foot and ankle trauma to the ED in the pre-pandemic period, during the COVID-19 emergency, and in the post-pandemic period. This study aims to assess epidemiological and clinical data. **Methods**: Five years of data were collected on admissions to the ED at the Fondazione Policlinico Universitario A. Gemelli using an electronic database. The system allowed the use of a numeric code assessed at triage ranging from 1 to 5, where 1 indicated a patient with compromised vital functions, and it continued in decreasing criticality. Data were extensively analyzed and extrapolated to obtain epidemiological and clinical evaluation. **Results**: Data from 3787 patients, including 1945 males with a mean age of 41.4 years, were collected. Data were evaluated in the three different periods. In the pre-pandemic period, 2228 ED admissions were recorded, including 1138 males with a mean age of 37.4 years and a mean of 2.79 admissions per day. Codes 3 and 4 reported in the ED triage were 4.8% and 90.1%, respectively; the average surgical treatment was 5.6%. During the COVID period, the total number of admissions was 981, with 501 males with an average age of 43.8 years and a mean of 1.30 admissions per day. Codes 3 and 4 were 22.5% and 72.7%, respectively; the average surgical treatment was 10.4%. In the post-COVID period, 578 admissions were, including 306 males with a mean age of 43.2 years, and a daily access rate of 1.58 patients. Codes 3 and 4 reported in the ED triage were 25.4% and 70.8%, respectively; the average surgical treatment was 8.6%. **Conclusions**: During the pandemic period due to COVID-19 a substantial decrease in total admissions per day in the ED was noted, but an increase in more complex codes occurred, as evidenced by the percentage increase in surgical admissions compared to total admissions during the pandemic; the mean age of users gradually increased. Total hospitalization data remained stable in the post-pandemic period, likely due to the global impact of the pandemic. COVID-19 radically and concretely changed people’s living habits and priorities for accessing the ED.

## 1. Introduction

Emergency Department (ED) visits for foot and ankle trauma cover a wide range of injuries, reflecting the susceptibility of these areas to impact and strain. Foot and ankle injuries are among the most common in emergency settings due to their complex anatomy and critical role in mobility [1,2].

According to Court-Brown et al. in the UK, foot and ankle fractures rank among the five most common bone fractures and are second only to femur and wrist fractures in Sweden [1,3]. In Australia, they are the leading cause of ED admissions, significantly impacting the healthcare system economically [4].

A United States study observed 673,214 ankle fractures over five years, with an average patient age of 37, and a prevalence of 56% in females [5]. Ankle fractures are associated with high incidence and complication rates, including chronic pain in over one-third of cases, posing a substantial burden on patients and healthcare payers [6].

In 2010, Tarantino et al. stated that in Italy, ankle fractures represent the third cause of hospitalization in the orthopedic field after hip and humerus fractures, with approximately 31.3% out of 29017 cases observed in 3 years [7].

A significant number of ED admissions for foot and ankle injuries stem from sports, with younger populations more prone to sprains, fractures, and ligament issues due to high-impact activities [8,9]. Workplace injuries are common in physically demanding jobs like construction (foot = 8.14%, ankle = 8.68%) and transportation (foot = 11.06%, ankle = 13.80%), with older workers experiencing fewer but more severe ankle injuries [10]. Traffic accidents also cause severe foot and ankle trauma, often involving complex fractures that may require surgical intervention and risk long-term impairments without prompt treatment [11,12].

In December 2019 and January 2020, a new coronavirus was identified in Wuhan, China in patients with pneumonia, and was confirmed through genome sequencing, PCR tests, and cultures [13,14]. It was named “COVID-19” by the WHO and its high contagiousness caused significant hospitalizations and mortality, prompting global governments to impose strict measures. Italy initiated a nationwide lockdown from 9 March to 4 May 2020, under Law Decree n° 59 (8 March 2020) [15], restricting movement, suspending non-essential activities, and implementing social distancing. Exceptions included healthcare personnel, pharmacies, and grocery stores, while healthcare workers’ leaves were suspended. Subsequent phases gradually eased restrictions. The national emergency ended on 31 March 2022, after the pandemic deeply affected daily life, the economy, and healthcare systems [16]. People’s daily lives, the economy, and of course healthcare providers were severely shattered by the COVID-19 pandemic [17,18].

During the various periods of closure and restrictions enforced in response to the COVID-19 pandemic, a significant and rapid decrease in ED admissions was observed across healthcare facilities. This decline was not uniform; it was accompanied by notable differentiation in the severity of injuries presented to emergency departments. Patients who sought care tended to exhibit more serious conditions, suggesting that many individuals delayed seeking medical attention for less severe issues due to fears of contracting the virus, potential exposure in healthcare settings, or the perception that their injuries were not urgent. These behavioral patterns posed significant challenges to the healthcare system, exacerbating the progression of chronic diseases and leading to increased morbidity and mortality among individuals with high-risk conditions.

This study aims to conduct a comprehensive retrospective analysis of all patients presenting to our emergency department during these tumultuous times. We aim to systematically examine the type and severity of injuries sustained by patients and the number of patients treated surgically at various times, and to gain a deeper understanding of the changes in patient demographics and the nature of foot and ankle injuries that occurred not only during the initial lockdown but also during subsequent phases of the pandemic response.

To achieve this, we will compare data collected during the pandemic with ER admissions before the onset of COVID-19. Specifically, the study will analyze patient records from 1 January 2018 to 8 March 2020, an interval that serves as a benchmark for typical patient presentations and treatment patterns in our department. In parallel, we will examine the recovery phase, defined as the period from 1 April 2022 to 1 April 2023, which reflects the transition to post-pandemic norms and the resumption of regular healthcare operations.

This retrospective analysis is significant not only for its immediate clinical implications but for its contribution to the existing body of literature as well. To the best of the authors’ knowledge, no articles have specifically studied the changes in the types of patients presenting with foot and ankle trauma throughout the year following the onset of the first COVID-19 quarantine. By analyzing the differences in injury patterns, severity, and treatment protocols during these critical periods, we hope to provide insights that may inform future emergency response strategies, improve trauma care pathways, and enhance patient outcomes in similar public health emergencies. Moreover, understanding these changes can help shape preventive strategies and inform healthcare policy, ensuring that the lessons learned from the pandemic are not lost and can be applied to improve the resilience of our healthcare systems in the face of future challenges.

## 2. Materials and Methods

The search was conducted retrospectively with the ED’s electronic database using the ICD-9 [19] diagnosis codes in the discharge report. The International Classification of Diseases, Ninth Revision, Clinical Modification (ICD-9-CM) is based on the Ninth Revision of the World Health Organization’s International Classification of Diseases (ICD-9). ICD-9-CM is the official system for assigning codes to diagnoses and procedures associated with hospitals throughout Italy.

The ICD-9-CM consists of the following:

- A tabular list containing a numerical list of the disease code numbers in tabular form;
- An alphabetical index of the disease entries;
- A classification system for surgical, diagnostic, and therapeutic procedures (alphabetic index and tabular list).

The pre-pandemic period (1 January 2018 to 8 March 2020), COVID-19 emergency (9 March 2020 to 31 March 2022), and post-pandemic period (1 April 2022 to 1 April 2023) were analyzed. Data were manually extracted and reviewed by the authors.

For each patient, the following data were included:

- Age;
- Sex;
- Triage severity code: increasing from 1 to 5;
- Prognosis days;
- Cause of injury: domestic, work (including school), traffic, sports, other (including self-injury, unspecified);
- Type of injuries;
- Whether the patient underwent surgery for the injury that led to the ED;
- Daily ED admissions.

### Statistical Analysis

Mean and standard deviation (SD) were used as quantitative variables. Welch’s *t*-test with a 95% confidence level was used to determine whether there was a statistical difference between the means obtained in the three periods considered. Using Pearson’s Chi-squared test, the evolution of the distribution of the following categories was compared: severity code; cause of injury; surgery; and type of trauma.

## 3. Results

### 3.1. Access to ED

During the pre-pandemic period spanning from 1 January 2018 to 8 March 2020, a total of 2228 people approached our ED with a daily average of 2.79 people per day. Starting from 9 March 2020, the beginning of the national lockdown, for the next two years until 31 March 2022, a decrease in ED visits was recorded, with 981 people and an average of 1.30 people per day. In the last phase, post-COVID, from 1 April 2022 to 1 April 2023, 578 individuals accessed our ED with an average of 1.58 people per day. Statistical analysis reveals a significant difference between the pre-COVID and COVID periods (*p* < 0.01), as well as between the pre-COVID and post-COVID phases (*p* < 0.01). However, the comparison between the COVID and post-COVID phases shows no significant difference, suggesting that, while visits have increased slightly since the peak of the pandemic, they have not returned to pre-pandemic frequencies (Table 1).

### 3.2. Sex and Age

The sex distribution of patients who accessed our ED remained consistent over the three periods analyzed, indicating no significant change. However, there remains a clear prevalence of male patients across the three periods (51.1 in both the pre-COVID era and the COVID era, and 52.9 in the post-COVID phase). The mean age was recorded to be significantly lower in the pre-COVID period. When compared with the next two periods, it was noted that there was an upward trend in mean age. The analysis showed a statistically significant relationship between the pre-COVID and COVID phases (*p* < 0.01). On the other hand, no significant difference between the COVID and post-COVID periods was found (Table 1).

### 3.3. Severity Codes and Prognosis Analysis

The severity of the patient’s clinical conditions was assessed using the severity codes given upon arrival at the ED and by the prognosis days recorded at their dismissal. Analysis revealed that during the COVID period, admissions to the ED appeared more severe by the severity code calculation and the increase in total prognosis days. Mean prognosis days increased from 14.24 in the pre-COVID period (SD: 9.71–range: 0–77) to 16.29 during the COVID period (SD: 10.16–range: 0–50). This appears to be statistically significant (*p* < 0.01). Mean prognosis during the post-COVID period appears to be consistent with the COVID period 16.19 (SD: 10.06–range: 0–40). Indeed, there is no sign of a statistical difference between the COVID and post-COVID period (*p* = 0.57) (Table 1).

**Figure 1:**
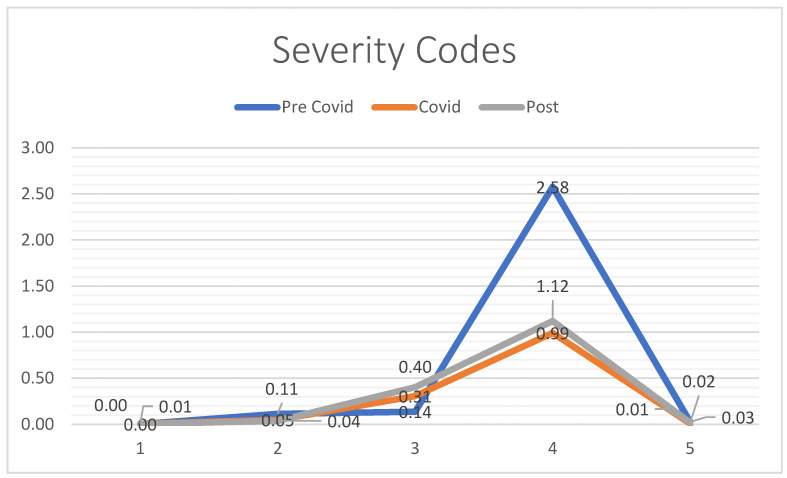
Severity codes (per-day).

The difference in severity code distributions per day was statistically significance (*p* < 0.01) between the pre-COVID and COVID periods. A noticeable upward trend was noted in code 3 ED admissions indicating a shift toward more moderate-to-severe cases observed in the ED; consistently, the severity of code 4 evaluations decreased. The post-COVID period appeared to align closely with the COVID period, resulting in what appears to be a statistically overlapping distribution of the severity codes (*p* = 0.34) (Figure 1).

### 3.4. Cause of Injuries

**Figure 2:**
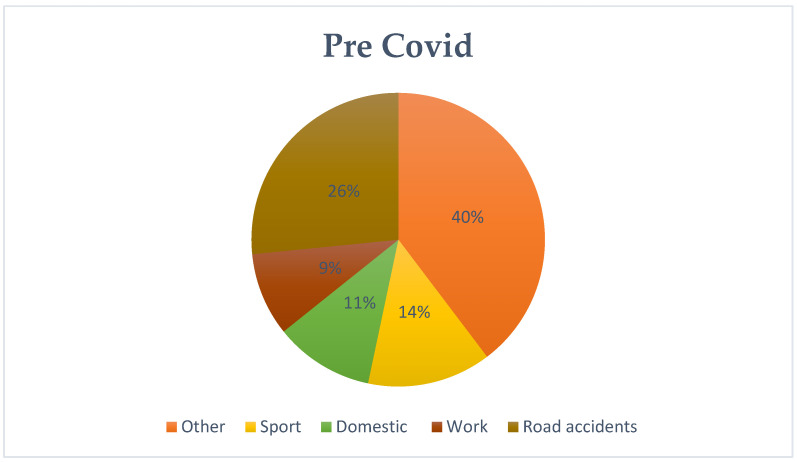
Pre COVID Causes of injury.

**Figure 3:**
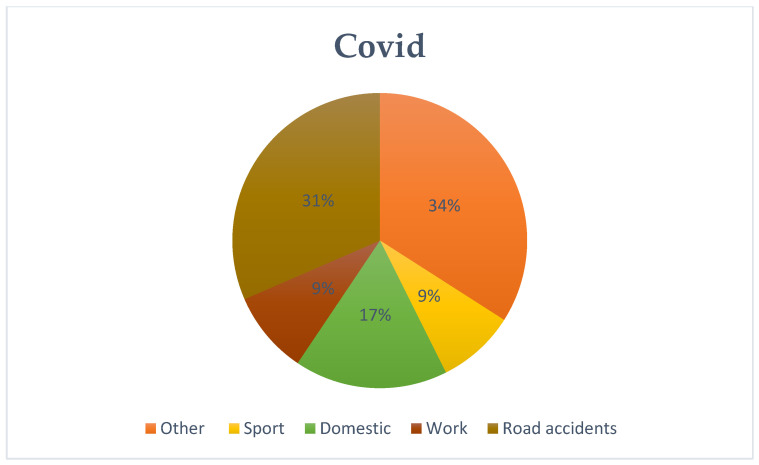
COVID Causes of injury.

**Figure 4:**
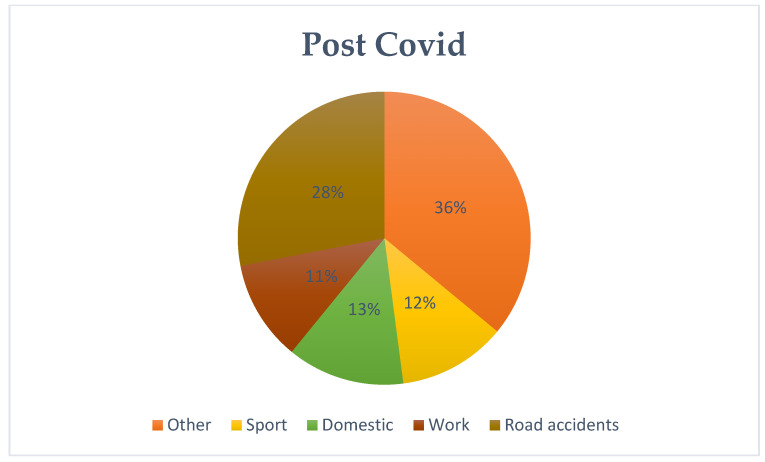
Post COVID Causes of injury.

An in-depth analysis of patients’ medical histories allowed us to categorize the causes of injuries which included, occupational, traffic, domestic (refers to injuries that occurred in the home environment, with commonly used utensils or tools for domestic use), and sports injuries. When a patient’s medical history lacked sufficient detail, the code “other” was used. The first interesting finding was the increasing trend in traffic-related accidents between the pre-COVID and COVID periods. This trend seems to return to pre-pandemic levels in the evaluation of post-COVID patient histories (pre-COVID: 26%–COVID: 31%–post-COVID: 28%). The analysis also highlighted a shift in sports-related injuries. Between the pre-COVID and COVID periods, a decrease was initially recorded and then the levels returned to pre-COVID levels after the end of the pandemic emergency (pre-COVID: 14%–COVID: 9%–post-COVID: 12%). Household accidents showed a different pattern during the pandemic due to the implemented restrictions (pre-COVID: 11%–COVID: 17%–post-COVID:13%); while work accidents exhibited relative stability (pre-COVID: 9%–COVID: 9%–post-COVID: 11%). Statistical analysis confirmed significant differences in the distribution of events between the pre-COVID and COVID periods (*p* < 0.01), but no significant change after the pandemic (*p* = 0.42). These patterns offer insight into the shifting sources of injury across the three periods and are summarized in Figure 2, Figure 3 and Figure 4 (Table 2).

### 3.5. Type of Injuries and Average Surgery

Overall, the most observed injuries in our ED were sprains and fractures. The term “other” was used to categorize other types of injuries. Looking at the difference in the types of injuries, it is possible to verify that significant differences were identified in the distributions of injury types, indicating that each period experienced a distinct pattern (*p* < 0.01). The three periods considered follow different statistically significant distributions (*p* < 0.01). The average number of patients operated on during the three periods follows a trend of increasing code severity during the pandemic. In fact, despite the decrease in ED admissions, the percentage of surgical treatments increased during the pandemic and the post-COVID period (Table 3).

## 4. Discussion

The COVID-19 pandemic profoundly impacted society, particularly healthcare delivery. Healthcare professionals faced unprecedented challenges, rapidly adapting to evolving roles and tasks to manage patient surges, reallocate resources, and mitigate virus transmission risks. Orthopedic care experienced significant changes, notably in elective foot and ankle procedures, which were largely deferred to prioritize critical COVID-19 care and limit non-urgent admissions. Trauma-related orthopedic cases also declined due to reduced injuries from mobility restrictions and social distancing measures.

In our experience, we observed a significant reduction in the overall number of foot and ankle trauma cases during the COVID-19 period. Interestingly, this reduction in trauma cases coincided with other demographic and clinical changes. For example, we recorded an increase in the average age of patients presenting with foot and ankle injuries, likely reflecting changes in social behavior and mobility patterns during lockdowns. In addition, we noted an increase in the severity of admission codes, indicating that patients who sought care had more complex or advanced conditions that required immediate intervention. The most relevant data on injury types highlighted decreased sports-related injuries and an increase in home-related injuries. This evidence suggests that sports activities were suspended or significantly discouraged during the pandemic. In contrast, household activities, including DIY projects, experienced a significant increase, even among individuals with limited experience in such areas, directly contributing to the rise in home-related injuries.

This shift in demographics likely reflects changes in risk perception and behaviors of different age groups during this period; younger populations may have been more willing to delay care, while older individuals with potentially more serious health problems continued to seek early treatment. It is useful to recall how regional and national policies were very harsh in discouraging outdoor recreational activities, even implementing administrative penalties, and how they tended to advise the elderly population to avoid going out except for strictly necessary or urgent reasons.

This trend underscores the broader impact of delayed or deferred care during the pandemic and the potential risks associated with deferring medical care. To minimize delays in medical and surgical care, our hospital created standardized and isolated pathways for patient admissions with COVID-19 diagnoses or symptoms attributable to it. The admission system was contingent on access to the ED, which was in a separate wing with dedicated pathways. In addition, confirmed COVID-19 cases were treated surgically in appropriately arranged operating rooms by select personnel. An important aspect of our results was the observation that the trends in the data regarding the number of cases, patient demographics, and the severity of lesions were maintained even in the year following the official end of the pandemic. This continuity suggests that the effects of the pandemic may have triggered a lasting change in patient behavior and health-seeking patterns, potentially leading to more cautious approaches to healthcare utilization. It also highlights the ongoing impact of the pandemic on healthcare delivery and the need for systems to adapt to potential changes in demand and resource deployment.

Although this study analyzes the largest dataset of its kind in Italy, the results may not fully represent trends across the entire metropolitan area of Rome. However, as a Level 1 trauma center, our hospital serves urban and rural populations beyond Rome, collecting diverse trauma cases. This broader catchment area provides insights applicable to other regions with similar high-volume trauma centers. While not reflecting all facilities in Rome, the findings highlight patterns relevant to comparable healthcare networks.

Some authors have published their experiences on this topic, focusing on shorter periods. It is certainly unequivocal that the total number of patients admitted to the ED for foot and ankle trauma decreased during the COVID period [8,20,21,22,23].

A significant finding in line with our results in White’s [8] experience is the increase in the mean age of patients arriving at the emergency department and the percentage increase in total admissions compared with the prior year (18.2% vs. 12.5%). A major reduction in total admissions (62%) was also confirmed.

In Khan’s experience [20], UK hospitals reported a halt in 91% of all elective surgeries, and 70% of patients reported that trauma surgeries were also stopped. Fifty-five percent of elective clinics had completely canceled lists; most importantly, only 9% of trauma centers operated normally, and 69% had reduced service.

In the United States, national estimates of emergency department admissions calculated from 2019 to 2021 showed 185,118 ankle fracture admissions with a 15% reduction in the post-COVID period in 2020. An important difference with our data was the prevalence of female sex, which increased by 1.3% during the first phase of the pandemic and by 1.1% in the second year of the pandemic. The average total age of admission remained consistent with our data [21].

Nath et al. [22] show a marked reduction in activity, with the incidence of foot and ankle fractures decreasing during the first lockdown. In contrast, they present results with an increasing incidence of fractures and surgeries performed in the second lockdown and a higher average pre-pandemic age than during the lockdown period. It is also useful to see that in a country like Sweden where restrictions were significantly less than in Italy, a reduction in hospital admissions for ankle fractures occurred, especially for women aged 70 years and older. The greatest reduction in fracture incidence occurred during the first 30 days of lockdown, evidence of greater adherence to social distancing rules [24].

Mason et al. [23] demonstrated that there were significant reductions in all foot and ankle injury cases during the COVID-19 pandemic in the UK from March to August 2020. National surgical activity was significantly reduced for all cases during the lockdown, however post-lockdown there was a normalization of activity in trauma and diabetic surgery with less than a quarter of elective activity having resumed by the end of the study.

Conversely, the number of surgical patients admitted increased on average with more serious codes. In Stringer’s experience, it is evident that the total number of trauma admissions decreased dramatically during the lockdown period. In fact, in the statistical analysis, they present a reduction in foot and ankle trauma between pre- and during COVID from 15.20% to 8.81% of all admissions for trauma to their ED [25].

The only group that showed discordant data were that of Shah et al. [26]. Their data presented a reduction in the mean age of patients compared with pre-pandemic year data. However, an increase in surgical procedures in the 2020 block phase was confirmed, which is a sign of ED admissions with higher mean severity. It must be said that this data was presented in a limited temporary distribution. In confirmation with our results, a survey of all German trauma centers showed a reduction in diagnoses requiring treatment in hospitals of lower levels of care. In contrast, there was evidence of a higher concentration of more serious diagnoses in level 1 trauma centers [27,28].

Several authors globally have published adaptations of their ankle and foot departments in response to the pandemic; the implementation of technologies, and in particular telemedicine, has been evaluated and successfully used for follow-ups, and the creation of COVID teams and dedicated pathways [29,30,31,32]. Interestingly, the telemedicine protocol drafted by Sa et al. [33] was based on three different phases and included a preparation for the visit with a phone call assessment and an explanation of the rules to make the teleassessment effective. A telehealth visit was divided into 12 steps and was developed to provide the patient with consistent quality and effective diagnosis; the 12 steps include: a detailed history; an inspection and gait examination; and the formulation of a list of differential diagnoses for most patients. Then, the patient was asked to perform maneuvers and movements to test and verify the formulated diagnoses; a follow-up phase followed in which at the end of each visit, patients who were candidates for surgery were able to schedule an appointment with the surgeon’s office before surgery to further discuss surgical planning. For postoperative and nonoperative managed patients, a traditional appointment was offered if deemed necessary by the virtual visit (e.g., to assess wound complications). However, for the vast majority of patients, follow-up was scheduled as a repeat telemedicine visit, given the limitations posed by the pandemic. It is useful, however, to recall Manz et al. [34], in which patients were more satisfied with outpatient visits than with telemedicine visits, although the vast majority of patients said they were willing to use telemedicine in the future.

## 5. Conclusions

During the COVID-19 pandemic, emergency room (ER) dynamics changed significantly due to public health measures, risk averse patients, and excessive demands on critical care resources. A substantial decrease in total daily ER admissions was observed as many people postponed or avoided nonemergency visits to reduce the risk of infection. This led to a reduction in overall ER visits, a trend seen in health facilities globally. However, while the total number of visits for foot and ankle trauma has decreased, there has been a noticeable shift in the severity of cases presenting to the ER, as the admission rates for patients with more serious conditions have increased. This was reflected in the increase in high-severity hospitalization codes, especially code 3; an indication that those who came to the ER often needed immediate and complex medical and surgical interventions.

Evidence of this change was found in the percentage increase in surgical treatments performed during the pandemic period, in sharp contrast to data from the pre-pandemic era.

Patients who previously might have received preventive or early-stage treatment in less acute settings were instead arriving with advanced conditions requiring urgent surgery. This trend is indicative of how the pandemic altered not only access to care but also the trajectory of patient outcomes, particularly for those with chronic or degenerative conditions that could have worsened during the waiting period. Another important observation was the gradual increase in the average age of patients presenting to emergency rooms during the pandemic. Data on total hospitalization rates and the percentage of patients who required surgical treatment showed surprising consistency when comparing the post-pandemic period with the pandemic. This continuity suggests that the influence of the pandemic on patient behavior and hospital resource utilization may have lasting effects, potentially marking a long-term change in how patients seek emergency services.

Thus, the COVID-19 pandemic left a profound impact not only on the daily lives and priorities of people accessing emergency rooms but also on the broader field of orthopedic surgery. It has prompted a reevaluation of treatment protocols and approaches for various conditions, reshaping the orthopedic surgical field’s response to trauma and chronic disease. These changes highlight the lasting legacy of the pandemic on healthcare access, patient behavior, and clinical decision-making.

## Figures and Tables

**Table 1:** Periods Comparision (P1: pre-COVID vs. COVID; P2: Pre-COVID vs. post-COVID; P3: COVID vs. post-COVID).

	Pre COVID	COVID	Post COVID	*p* Value
Total number of patients	2228	981	578	
Average access per day	2.79	1.30	1.58	P1 (*p* < 0.01)–P2 (*p* < 0.01) P3 (*p* = 0.11)
Male	1138 (51.1%)	501 (51.1%)	306 (52.9%)	
Female	1090 (48.9%)	480 (48.9%)	272 (47.1%)	
Mean age of Patients	37.4	43.8	43.2	P1 (*p* < 0.01)–P2 (*p* < 0.01) P3 (*p* > 0.05)
(Range)	(1–101)	(4–96)	(7–97)	
(Standard Deviation)	20.8	17.5	16.8	
Mean prognosis days	14.24	16.29	16.19	P1 (*p* < 0.01)–P2 (*p* < 0.01) P3 (*p* = 0.57)
(Range)	(0–77)	(0–50)	(0–40)	
(Standard Deviation)	9.71	10.16	10.06	

**Table 2:** Cause of injuries.

Periods	*p*-Values
Pre-COVID/COVID	*p* < 0.01
COVID/Post COVID	*p* = 0.42

**Table 3:** Type of injuries.

	Pre-COVID	COVID	Post-COVID
Sprain/Dislocation	1246 (55.9%)	456 (46.5%)	283 (49.0%)
Fractures	509 (22.8%)	313 (31.9%)	139 (24.0%)
Other	473 (21.2%)	212 (21.6%)	156 (27.0%)
Tot. Surgery	126 (5.6%)	102 (10.4%)	52 (8.6%)

## Data Availability

The study data will be available upon request to the corresponding author (email: chiara.comisi22@gmail.com).
